# Exploring the role of circulating proteins in multiple myeloma risk: a Mendelian randomization study

**DOI:** 10.1038/s41598-025-86222-5

**Published:** 2025-01-30

**Authors:** Matthew A. Lee, Kate L. Burley, Emma L. Hazelwood, Sally Moore, Sarah J. Lewis, Lucy J. Goudswaard

**Affiliations:** 1 Nutrition and Metabolism Branch, International Agency for Research on Cancer, World Health Organisation, Lyon, France; 2 Population Health Sciences, University of Bristol, Bristol, UK; 3 MRC Integrative Epidemiology Unit, University of Bristol, Bristol, UK; 4 University Hospitals Bristol and Weston NHS Foundation Trust, Bristol, UK; 5 School of Cellular and Molecular Medicine, University of Bristol, Bristol, UK

**Keywords:** Proteomics, Multiple myeloma, Mendelian randomization, Genetic colocalization, Myeloma, Genetics

## Abstract

Multiple myeloma (MM) is an incurable blood cancer with unclear aetiology. Proteomics is a valuable tool in exploring mechanisms of disease. We investigated the causal relationship between circulating proteins and MM risk, using two of the largest cohorts with proteomics data to-date. We performed bidirectional two-sample Mendelian randomization (MR; forward MR = causal effect estimation of proteins and MM risk; reverse MR = causal effect estimation of MM risk and proteins). Summary statistics for plasma proteins were obtained from genome-wide association studies performed using SomaLogic (N = 35,559; deCODE) and Olink (N = 34,557; UK Biobank; UKB) proteomic platforms and for MM risk from a meta-analysis of UKB and FinnGen (case = 1649; control = 727,247) or FinnGen only (case = 1085; control = 271,463). *Cis-*SNPs associated with protein levels were used to instrument circulating proteins. We evaluated proteins for the consistency of directions of effect across MR analyses (with 95% confidence intervals not overlapping the null) and corroborating evidence from genetic colocalization. In the forward MR, 994 (SomaLogic) and 1570 (Olink) proteins were instrumentable. 440 proteins were analysed in both deCODE and UKB; 302 (69%) of these showed consistent directions of effect in the forward MR. Seven proteins had 95% confidence intervals (CIs) that did not overlap the null in both forward MR analyses and did not have evidence for an effect in the reverse direction: higher levels of dermatopontin (DPT), beta-crystallin B1 (CRYBB1), interleukin-18-binding protein (IL18BP) and vascular endothelial growth factor receptor 2 (KDR) and lower levels of odorant-binding protein 2b (OBP2B), glutamate-cysteine ligase regulatory subunit (GCLM) and gamma-crystallin D (CRYGD) were implicated in increasing MM risk. Evidence from genetic colocalization did not meet our threshold for a shared causal signal between any of these proteins and MM risk (h4 < 0.8). Our results highlight seven circulating proteins which may be involved in MM risk. Although evidence from genetic colocalization suggests these associations may not be robust to the effects of horizontal pleiotropy, these proteins may be useful markers of MM risk. Future work should explore the utility of these proteins in disease prediction or prevention using proteomic data from patients with MM or precursor conditions.

## Introduction

Multiple myeloma (MM) is the second most common haematological malignancy in the UK, with ~ 6000 new cases each year^1^. It is characterised by neoplastic proliferation of plasma cells in the bone marrow, resulting in overproduction of monoclonal immunoglobulins commonly referred to as paraprotein or M-protein. Nearly all cases of MM are preceded by the benign asymptomatic precursor condition, monoclonal gammopathy of unknown significance (MGUS)^2,3^. The diagnosis of active MM is based on the presence of M-protein in serum or urine, along with evidence of end-organ damage including hypercalcemia, renal insufficiency, anaemia, and bone lesions^4^. Patients with MM are at an increased risk of developing blood clots and are susceptible to recurrent and severe infections^5–7^. Periods of disease remission can be induced using chemotherapy regimens including proteosome inhibitors (e.g. bortezomib), immunomodulatory drugs (e.g. lenalidomide), and monoclonal antibodies (e.g. daratumumab), plus autologous stem cell transplant for a subset of eligible patients^8^ However, MM is currently incurable with a median overall survival of 6 years^8^. The pathogenesis of MM is not fully understood, potentially limiting the identification of curative therapeutic strategies.

**Fig. 1:**
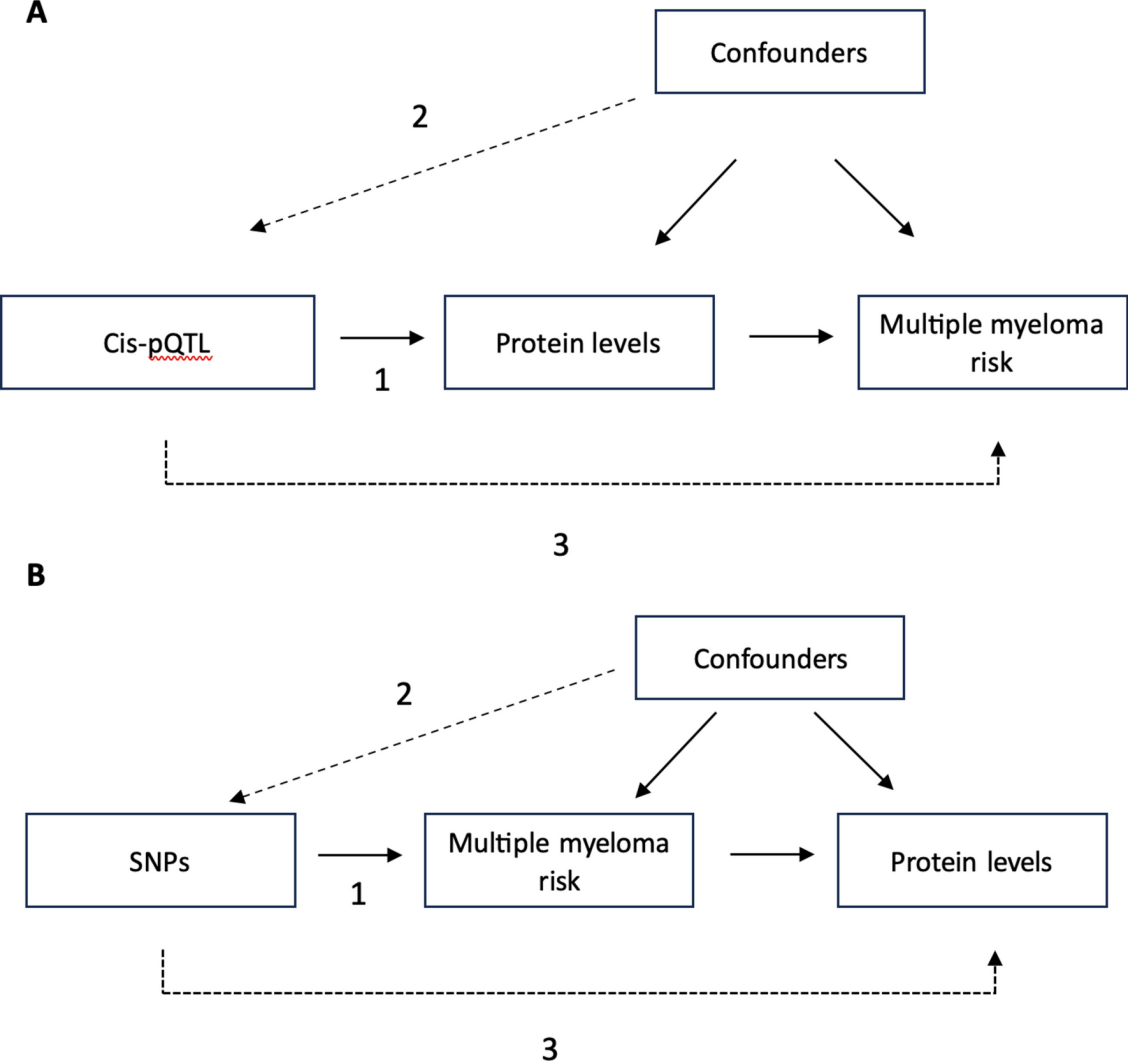
Directed acyclic graph of bidirectional Mendelian randomisation analyses. (**A**) The effect of circulating proteins on multiple myeloma risk. (**B**) The effect of multiple myeloma risk on circulating proteins. The Mendelian randomization assumptions are given as 1–3: (1) the genetic variant is robustly associated with the exposure; (2) there are no confounders of the genetic variant and outcome association; (3) the genetic variant is associated with the outcome only via its association with the exposure. SNPs: single nucleotide polymorphism; pQTL: protein quantitative trait loci (SNPs associated with the abundance of a protein).

Observational studies have reported multiple risk factors associated with the development of MM, including age, sex, ancestry, family history, and adiposity^9–14^. However, observational studies are often limited by reverse causation (for example, where an association between exposure and outcome is driven by prevalent outcome influencing the exposure) and confounding (for example, where an exposure-outcome association is spuriously identified due to the association between a third trait (confounder) with both the exposure and outcome)^15^. Mendelian randomization (MR) is an approach which uses genetic variants (alleles randomly assigned during gametogenesis; typically, single nucleotide polymorphisms (SNPs)) to estimate the causal effect of an exposure on an outcome^16–18^. When core assumptions are satisfied (Fig. 1; see statistical analysis section), MR is robust to biases observed in conventional observational analyses (e.g., reverse causation and confounding)^15,19^. Previous studies have used MR to investigate evidence for a causal role of known MM risk factors (such as obesity) in disease development and identified novel factors such as increased telomere length to be implicated in MM risk^20^.

Alterations in the abundance of specific circulating proteins has been used previously in cancer diagnosis and risk stratification^21^ and can also highlight mechanistic pathways^22,23^. There is evidence that the inclusion of proteomics in clinical prediction tools improves MM risk prediction in comparison to using clinical risk factors alone^24^. Furthermore, as proteins are the main target for small molecule drugs and biologics^25^, exploring the role of circulating proteins in MM risk using MR may identify targets for intervention. A recent MR study identified 13 potentially causal proteins^26^ but was limited in scale and may suffer bias due to horizontal pleiotropy (where the genetic variant has an effect on the outcome that is not via its effect on the exposure; Fig. 1). Horizontal pleiotropy may occur as authors included *trans* genetic variants (variants not in or near the gene coding region for the protein of interest)^27^. As a result, these *trans* genetic variants are more likely to be pleiotropic variants than *cis* genetic variants). Authors also did not use an independent sample to replicate results^26^. We set out to systematically explore the relationship between circulating proteins and MM risk using two-sample MR with robust instrument selection^15,19^ and corroborate our findings using genetic colocalization.

## Methods

### Overview

Using data for circulating proteins from two genome-wide association studies (GWAS), we performed bidirectional two sample MR analyses to explore the causal role of circulating proteins in MM risk (Fig. 1). We performed a forward MR to estimate the effect of circulating proteins on MM risk and a reverse MR to estimate the effect of MM risk on circulating proteins. The latter was performed to evaluate evidence for reverse causality, where MM may influence protein levels. We performed genetic colocalization to investigate shared causal signals between circulating proteins and MM risk, which may strengthen evidence for causal effects identified in the MR analyses or highlight non-causal markers of MM risk that warrant further investigation^28^. Data for circulating proteins were obtained from GWAS performed in two independent studies of European ancestry: deCODE (SomaLogic) and UK Biobank (Olink). Data for MM risk were obtained from GWAS performed in two independent studies (UK Biobank and FinnGen) which we meta-analysed. Given overlap of UK Biobank data (which is a source of bias in MR^29^), we used deCODE proteins with the meta-analysis of MM risk and UK Biobank proteins with the FinnGen GWAS^30,31^. All GWAS performed followed standard quality control procedures (for example, imputation quality score > 0.9 and minor allele frequency > 1%) to filter directly sequenced and imputed variants.

### Circulating protein GWAS

GWAS data for up-to 4907 aptamers (4719 unique proteins) were obtained from Ferkingstad et al.^32^. Protein concentrations were measured from ethylenediaminetetraacetic acid (EDTA) plasma samples from 35,559 Icelandic individuals (deCODE) using SomaScan® (SomaLogic). Briefly, a large proportion of the Icelandic population enrolled in a nationwide programme administered by deCODE genetics; 49,708 enrolled individuals underwent whole-genome sequencing while 166,281 additional individuals were genotyped with imputation based on the whole-genome sequencing data^32^. The SomaScan platform uses Slow Off-rate Modified Aptamers which make direct contact with proteins, enabling their detection and quantification in relative fluorescence units (RFUs) using a DNA microarray. Multiple aptamers can bind to a single protein (e.g., because of splice-isoforms). UK Biobank is a population-based cohort of ~ 500,000 individuals aged 40–69 recruited between 2006 and 2010 in the United Kingdom. Genotyping and imputation have been described previously^33^. Briefly, the genotyping and imputation involved a two-step imputation process performed first using the Haplotype Reference Consortium and then performed using a merged UK10K and 1000 Genomes Phase 3 reference panel, these two imputations were combined and the HRC imputed variant was kept in instances of duplication. Prior to genome-wide analysis, protein values were inverse rank normal transformed and adjusted for age, sex and sample age. These residuals were standardised again using an inverse rank normal transformation and a linear mixed-model (LMM) GWAS was performed using BOLT-LMM^32,34–36^. Assuming the distribution of protein concentration was normal prior to inverse rank normal transformation, we interpret these units to be approximately equivalent to a normalized standard deviation (SD).

Genome-wide summary level data were also obtained for up to 2923 proteins from Sun et al.^37^. Protein concentrations were measured from EDTA plasma samples from 34,557 participants of European ancestry from UK Biobank using the Olink Explore 3072 panel. Briefly, Olink Explore 3072 uses a proximity extension assay (PEA) which uses matched pairs of antibodies with DNA tags that, once bound to their target protein, hybridise and can be amplified and quantified using polymerase chain reaction. Proteins are measured in normalised protein expression (NPX) units which are on a log2 scale^38^. Prior to genome-wide analysis, protein values were inverse rank normal transformed and a whole-genome regression model using a leave one chromosome out scheme was performed with REGENIE (version 2.2.1)^37^ adjusting for age, age^2^, sex, age × sex, age^2^ × sex, batch, centre, genetic array, time between blood sampling measurement and the first 20 principal components. Assuming the distribution of protein levels was normal prior to inverse rank normal transformation, we interpret these units to be approximately equivalent to a normalized SD.

### Genetic instruments for circulating protein levels

In MR analyses of circulating proteins and MM risk we used *cis*-SNPs to instrument proteins. *Cis*-SNPs were obtained from the supplementary data of the original study. Briefly, a 1 mega base (1,000,000 bases; Mb) region was defined around each SNP reaching the genome-wide significance threshold specified in each GWAS (*p* value < 1.8 × 10^−9^ in deCODE; *p* value < 1.7 × 10^−11^ in UKB) which were ≤ 1 Mb from the transcription start site of the protein coding gene (discovery) or ≤ 1 Mb from the gene encoding the measured protein (replication). Starting with the SNP with the lowest *p* value, any overlapping regions were merged until no overlapping regions remained (major histocompatibility complex was treated as a single region). Linkage disequilibrium (LD) based clumping was used to identify whether regions were associated with multiple proteins; regional SNPs with high LD (r^2^ ≥ 0.8) were merged into a single region and the SNP with the lowest *p* value was considered the sentinel SNP. In total, 1192 of 4907 aptamers (deCODE) and 1860 of 2923 proteins (UKB) had available *cis*-SNPs.

### Multiple myeloma genome-wide association studies

For our MR analyses using deCODE SomaLogic protein GWAS as the exposure, genome-wide summary level data for MM risk were obtained from a meta-analysis of 1649 cases and 727,247 controls from two GWAS conducted in UK Biobank and FinnGen. In UK Biobank^39^, MM case status was recorded according to the International Classification of Diseases 10th revision (ICD 10)^40^ following mapping to Phecode v.1.2 (code 204.4 for MM)^41^. Cases were defined as those having the MM Phecode between recruitment (2006–2010) and the end of 2017; controls were defined as any individual who had not ever been diagnosed with MM (those who did not have the MM ICD 10 code). This gave a total of 564 MM cases and 455,784 controls at the time the GWAS was run. Models were adjusted for age, age^2^, sex, age × sex, age^2^ × sex and the top 20 PCs provided by UK Biobank. In FinnGen^42^ (cases = 1085; controls = 271,463), MM was recorded according to the International Classification of Diseases (ICD-O-3) following linkage with the Finnish Cancer Registers, controls were defined as any individual without any cancer diagnosis, and models were adjusted for sex, age, top 10 PCs, and genotyping batch. Meta-analysis was performed to increase the power of the MM GWAS using METAL (version 2011-03-25) and results were filtered to remove SNPs with a heterogeneity *p* value ≤ 0.05 between the two GWAS^43^. The METAL software was used to combine test statistics and standard errors and control for population stratification as recommended in the METAL documentation^44^. Estimates for each SNP indicates the difference in disease risk for each copy of the effect allele.

### Instruments for multiple myeloma risk

We used all SNPs which met the following requirement to instrument MM risk: a genome-wide significance threshold of *p* < 5 × 10^−8^ and an LD R^2^ threshold of 0.001 within a 10 kilo-base (kb) window to identify robust and independently associated SNPs. In the meta-analysis of MM GWAS, 1 SNP met the genome-wide significance threshold and was used to instrument MM risk. In the FinnGen MM GWAS no SNPs met the genome-wide significance threshold; instead, a lower threshold of *p* < 5 × 10^−7^ was used and 3 SNPs were identified. Of these, 2 SNPs (rs555992394 and rs8141529) were available in the UKB proteomic GWAS data. In relaxing the *p* value threshold for the MM GWAS in FinnGen, we may invalidate a core assumption of MR that the instrument is robustly associated with the exposure. As such, we caution that these analyses were employed to evaluate possible conflicting evidence to the forward MR and should not be interpreted as causal estimates of MM liability on protein levels. We also explored possible pleiotropic effects that may arise from this relaxation via searching the SNP rsIDs in the IEU Open GWAS Project^45^, essentially performing a phenome-wide association study (PheWAS).

### Statistical analysis

#### Mendelian randomization analysis

MR relies upon three core assumptions (Fig. 1): (1) the genetic variant is associated with the exposure, (2) there are no confounders of the genetic variant and outcome association (such as population structure), and (3) the genetic variant is associated with the outcome only via its association with the exposure (i.e., not via alternate pathways)^16^.

For all exposures, the following summary-level data were obtained from the original GWAS: rsID, effect allele, other allele, effect allele frequency (EAF), effect estimate, standard error of the effect estimate, *p* value of the effect estimate, and where available sample size for each SNP. Where individual SNP sample size was not available the overall sample size was used. Genetic variants were extracted from each outcome GWAS and, where these were not available, proxy SNPs were included if LD was ≥ 0.8. For all SNPs, the inclusion of SNPs where the reference strand was ambiguous was allowed and the reference strand was inferred using minor allele frequency (where minor allele frequency was not ≥ 0.3, in which case the proxy SNP was excluded). Data were harmonized such that the exposure effect allele was on the increasing scale. As such, MR estimates for the effect of circulating proteins on MM risk are given as the per effect allele normalised SD unit increase in protein concentration whereas estimates for the effect of MM risk on circulating protein levels are given as the normalised SD unit difference in protein per effect allele increase in disease risk. We used F-statistics to assess instrument strength, with an F-statistic > 10 indicating a strong instrument^46^. F-statistics were calculated as: F = R^2^ × (N − 1 − k)/((1 − R^2^) × k), where k is the number of SNPs in the instrument and N is sample size of the SNP-exposure GWAS. R^2^ was calculated as: R^2^ = (2(b2) × EAF ×  (1 − EAF))/((2(2) × EAF × (1 − EAF)) + ((SE2) × (2 × N) × EAF ×  (1 − EAF))), where b is the SNP-exposure association, EAF is the effect allele frequency of the SNP, SE is the standard error of the SNP-exposure association, and N is sample size of the SNP-exposure GWAS^47^. All exposure data are given in Supplementary Table 1 and Supplementary Table 2 for proteins and Supplementary Table 3 for MM.

For the forward MR analysis for circulating proteins and MM risk, two protein GWAS were used. Where proteins were measured by SomaLogic in deCODE, the MM GWAS used was the meta-analysis of FinnGen and UKB. Where proteins were measured by Olink in UKB, the MM GWAS was in FinnGen alone (to avoid sample overlap). To maximise power for MM risk, and given instrument strength for proteins was high^48^, we re-ran analyses for the UKB Olink proteins using the meta-analysis of the MM GWAS. Each protein was instrumented with a single *cis*-SNP^49^. As such, the Wald ratio^50^, which is the ratio of the SNP-outcome association divided by the SNP-exposure association, was used to estimate the effect of the protein on MM risk^50^. For the reverse MR, where there was a single SNP the Wald ratio was implemented, and where there were 2 or more (when instrumenting MM) an inverse variance weighted multiplicative random effects (IVW-MRE) model, which combines Wald ratios together in a meta-analysis, adjusting for heterogeneity^51^, was used as the primary model. The IVW-MRE model assumes that the strength of association of genetic instruments with the exposure does not correlate with the size of the pleiotropic effects and that the pleiotropic effects have an average of zero.

We performed Steiger directionality tests to assess whether the direction of effect being tested (either protein-MM risk or MM risk-protein) was supported^52,53^. The Steiger test calculates the variance explained in the exposure and the variance explained in the outcome by the exposure-related instruments. If more variance is explained in the outcome than the exposure, this may indicate a violation of MR assumption 3, that the genetic instrument is only associated with the outcome via the exposure^52^. When performing Steiger directionality tests with a single variant it can be difficult to distinguish between causal and pleiotropic models^52^. In such instances, and which we apply here, it is beneficial to combine evidence with that of bidirectional MR analyses and look for consistency. Proportion of variance liability was calculated for the UK Biobank MM GWAS using prevalence data for the United Kingdom and for the FinnGen MM GWAS using prevalence data for Finland. Prevalence data were obtained from the World Health Organization^54^. The 5-year prevalence of MM in the United Kingdom in 2022 was 1.4 per 100,000, and for Finland the prevalence was 1.1 per 100,000. These prevalence statistics were used to calculate a weighted prevalence for the meta-analysis of UK Biobank and FinnGen^55^.

#### Colocalization

We performed genetic colocalization analyses of all circulating proteins performed in the MR and MM risk. We extracted 125 kb, 250 kb, 500 kb and 1 Mb windows around the *cis*-SNP used in the MR analysis. We extracted all SNPs in these windows from the MM GWAS. We used the 1 Mb window as our main analysis and used the other windows to examine sensitivities to the number of SNPs included in the colocalization analysis. Signals present in multiple windows are unlikely to be driven by window size. Colocalization was implemented using the single causal variant assumption of Giambartolomei et al.^56^. The European population of the 1000 genomes reference panel (phase 3) was used to generate LD matrices. Priors were set based on 5000 SNPs^57^: *p*^1^ = 10^−6^, *p*^2^ = 10^−6^, and *p*^12^ = 10^−7^; where: p1 is the prior probability that a random SNP in the region is associated with the protein and not MM risk, p2 is the prior probability that a random SNP in the region is associated with MM risk and not the protein, and p12 is the prior probability that a random SNP in the region is associated with the protein and MM risk.

#### Identifying causal effects

We do not focus on *p* values in identifying potential causal relationships. Instead, we look for consistent evidence of effect in our two studies and use a multi-step approach to limit false positives: (1) the effect estimate was consistent in direction across both forward MR analyses, (2) the 95% CI did not overlap the null in both forward MR analyses, (3) a true direction of effect indicated by the protein-MM risk Steiger directionality test, and (4) there was no evidence for an effect in the reverse MR analysis. Furthermore, we consider evidence for a potential causal relationship to be strongest where there is also evidence from colocalization (h4 > 0.8).

## Results

### Causal effects of circulating proteins on MM risk

In our MR analyses using two independent protein GWAS to instrument protein levels, a total of 2564 proteins had suitable genetic instruments which were available in the outcome GWAS—994 proteins measured by SomaLogic and 1570 measured by Olink. 440 unique proteins (based on gene name) were measured and had instruments in both protein GWAS and 36% of these were instrumented by the same SNP. MR estimates for the forward MR are presented as odds ratios for MM risk, which are calculated for a per unit increase in protein. Results for all MR analyses are presented in Supplementary Table 4 and Supplementary Table 5.

**Fig. 2:**
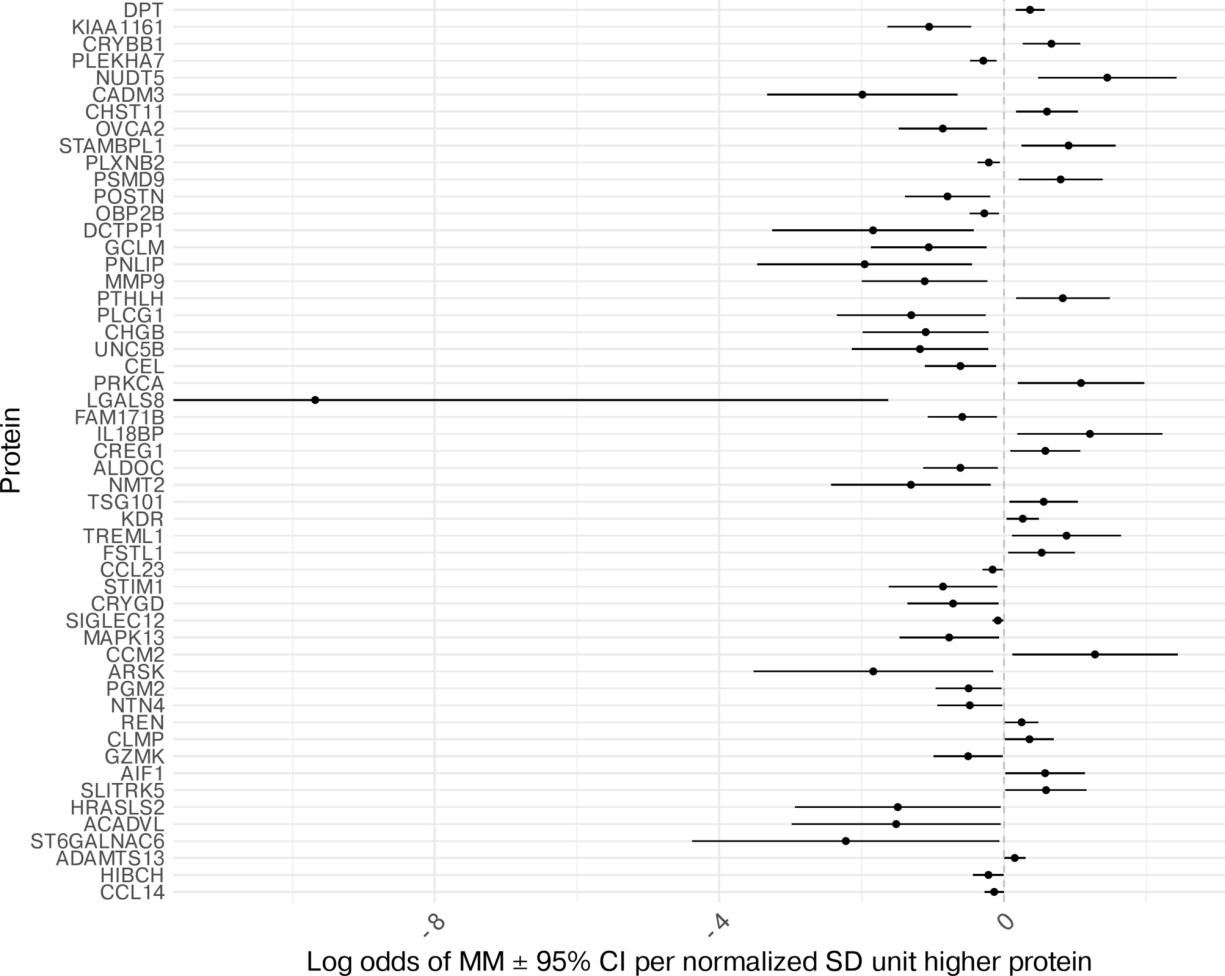
Estimates of the effect of circulating proteins (SomaLogic) on risk of multiple myeloma. Mendelian randomization analysis performed with protein genome-wide association study (GWAS) data from deCODE (SomaLogic) and outcome multiple myeloma data from meta-analysis of GWAS from UK Biobank and FinnGen. Proteins (Y axis) are represented by gene names (Additional File 1; Supplementary Table 4).

In the MR analysis using data from deCODE (SomaLogic) proteins as the exposure and the FinnGen/UKB meta-analysis of MM risk as the outcome, 53 circulating proteins had 95% CIs that did not cross the null (Additional File 1; Supplementary Table 4; Fig. 2). There was evidence that higher levels of proteins such as dermatopontin (DPT) and Beta-crystallin 1 (CRYBB1) had an increasing effect on MM risk (ORs per normalised SD unit of protein 1.44 (95% CI 1.18–1.77) and 1.95 (95% CI 1.30–2.92), respectively). For all 53 proteins, Steiger directionality tests suggested the tested direction was the true causal direction (Additional File 1; Supplementary Table 6). In the reverse MR, there was evidence that MM risk may impact levels of 1 of the 53 proteins: matrix metalloproteinase-9 (MMP-9; Supplementary Table 7).

**Fig. 3:**
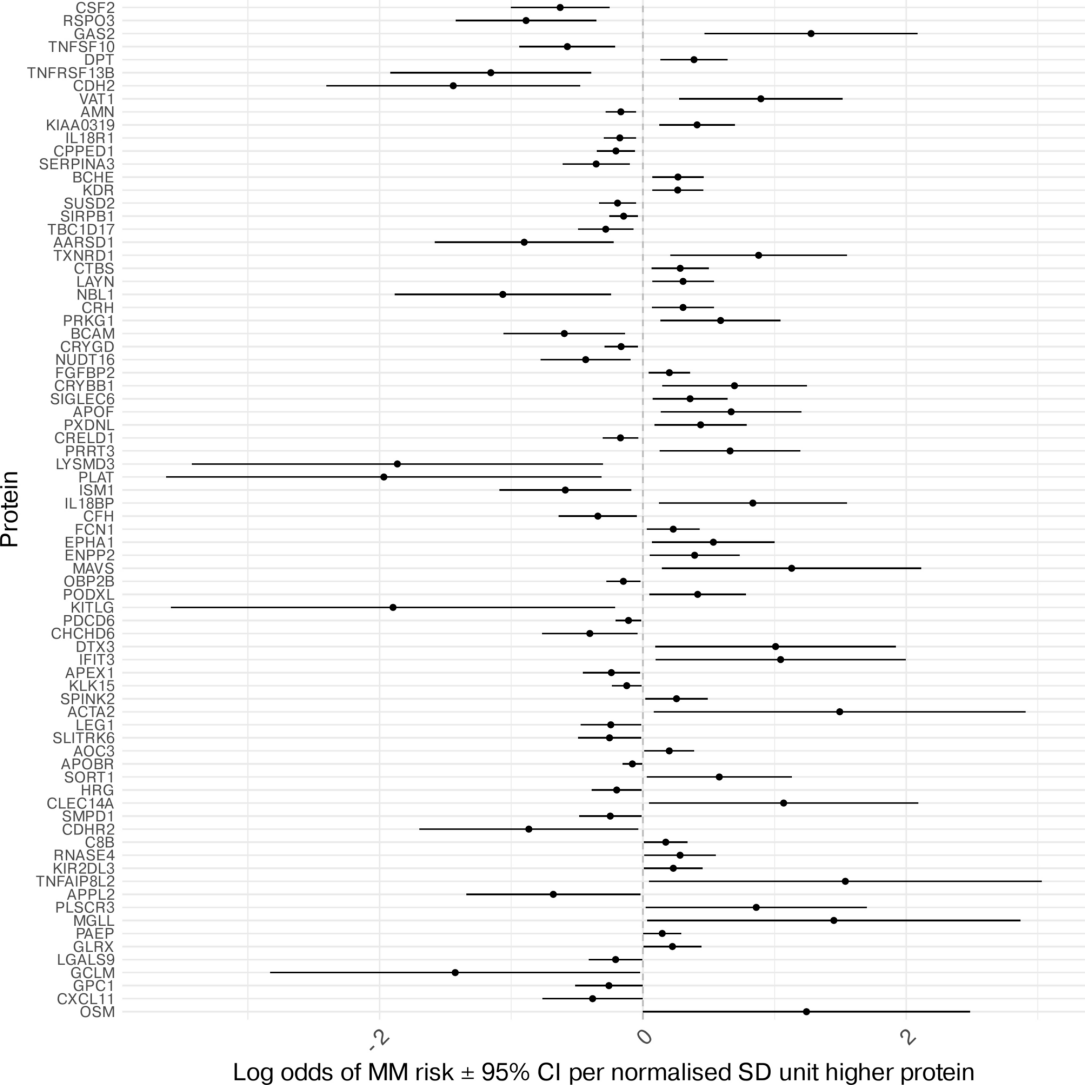
Estimates of the effect of Olink circulating proteins on risk of multiple myeloma. Mendelian randomization analysis using exposure protein genome-wide association study (GWAS) from UK Biobank (Olink) and outcome multiple myeloma GWAS data from FinnGen. Proteins (Y axis) are represented by gene names (Additional File 1; Supplementary Table 5).

In the MR analysis using data from UK Biobank (Olink) proteins as the exposure and FinnGen myeloma GWAS as the outcome, 78 circulating proteins had MR estimates with 95% CIs that did not cross the null (Additional File 1; Supplementary Table 5, Fig. 3) and results were concordant when using the MM meta-analysis (Additional File 1; Supplementary Table 8). For example, higher levels of proteins such as granulocyte–macrophage colony stimulating factor (CSF2, OR 0.53, 95% CI 0.37–0.78), R-spondin-3 (RSPO3, OR 0.41, 95% CI 0.24–0.70) and tumour necrosis factor ligand superfamily member 10 (TNFSF10, OR 0.56, 95% CI 0.39–0.81) decreased MM risk and higher levels of dermatopontin (DPT) increased MM risk (OR 1.47, 95% CI 1.14–1.90). For all 78 proteins, Steiger directionality tests suggested the tested direction was the true causal direction (Additional File 1; Supplementary Table 9). In the reverse MR, 1 of the 78 proteins (Odorant-binding protein 2b, OBP2B) had evidence for an effect of MM risk on protein levels (Additional File 1; Supplementary Table 10). Both SNPs used to instrument MM risk were also associated with amyloidosis (rs555992394) and had evidence for having an effect on blood cell counts including lymphocyte count and red cell distribution width (rs8141529) in the pheWAS analysis. These effects may be part of the shared causal pathway rather than pleiotropic given that amyloidosis and MM both share the precursor condition, MGUS^58^, and that MM is known to have an effect on blood cell counts through myelosuppression^59^.

**Fig. 4:**
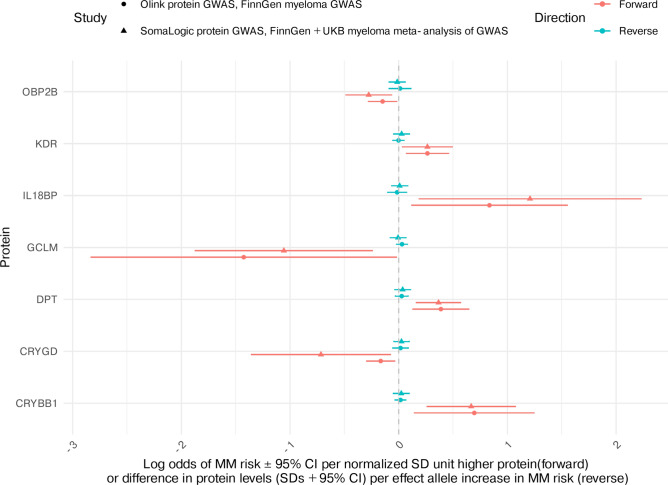
Effect of circulating proteins on multiple myeloma risk: consistent effects in Mendelian randomization analyses. Results are given for 7 proteins with consistent directions of effect, 95% confidence intervals (CIs) that do not cross the null, and no evidence of reverse effect across both MR analyses. Proteins (Y axis) are represented by gene names.

A total of 440 aptamers/proteins were shared across both MR analyses exploring the effect of circulating proteins on MM risk, therefore resulting in two MR estimates. For 157 of these aptamers/proteins, the cis-SNP identified for each from the Olink GWAS was also the cis-SNP identified from the SomaLogic GWAS. Where proteins were included in MR analyses using instruments from both protein GWAS, effect estimates from both forward MR analyses are available in Supplementary Table 11. Of these shared proteins, a total of 302 had consistent directions of effect (both negative or both positive beta coefficients) across MR analyses, seven of which had 95% CIs which did not overlap the null in both analyses. In the reverse MR, MM risk had little evidence for an effect on all seven of these circulating proteins (Fig. 4). Of these seven circulating proteins, an increase in abundance of four proteins (dermatopontin (DPT), beta-crystallin B1 (CRYBB1), interleukin-18-binding protein (IL18BP) and vascular endothelial growth factor receptor 2 (KDR)) was associated with an increase in MM risk, while an increase in the abundance of 3 proteins (odorant-binding protein 2b (OBP2B), glutamate-cysteine ligase regulatory subunit (GCLM) and gamma-crystallin D (CRYGD)) was associated with decreased MM risk.

### Colocalization

In colocalization analyses for the seven proteins with MR evidence for a causal effect on MM risk (Fig. 4), evidence to support colocalization was limited across all windows (h4 < 0.8 Supplementary Table 12, Supplementary Table 13). There was evidence for a shared causal variant between one protein, uncharacterized family 31 glucosidase KIAA1161, and multiple myeloma (h4 = 0.84 across all colocalization windows). This protein was detected and quantified in deCODE (SomaLogic), where there was MR evidence to support a causal relationship, but it was not measured in UKB (Olink).

## Discussion

In this study, bidirectional MR and genetic colocalization analyses were performed to identify whether circulating proteins causally impact MM risk. There was evidence using two protein GWAS that higher levels of four circulating proteins may increase MM risk and higher levels of three circulating proteins may decrease MM risk, however none of these results were supported by genetic colocalization, possibly indicative of low power or that estimates may not be robust to horizontal pleiotropy. A single protein, KIAA1161, measured only by SomaLogic, with evidence of an increasing effect on MM risk was supported by evidence from genetic colocalization.

Two previous MR studies have explored the effect of circulating proteins on MM risk. The first focused on inflammatory proteins alone^26^, whereas the second used GWAS data from protein levels measured by the SomaScan in a smaller sample (3301 participants) from the INTERVAL study^60^. Four of the 13 proteins with evidence for a causal relationship with MM risk by Wang et al. were also instrumented in our analysis. Our MR evidence (also using SomaScan) only supported a causal relationship for one of these proteins and MM risk (follistatin-related protein 1, FSTL1). We did not find evidence for a causal effect for the other three proteins, this may be due to the use of *trans* SNPs by Wang et al. which likely included pleiotropic pathways^60^.

All seven proteins with consistent evidence across our two protein datasets have limited evidence in the literature of having previously been implicated in the pathogenesis or progression to MM^61^. The strongest evidence for an effect was with dermatopontin on MM risk, where higher levels were associated with an increase in MM risk. DPT is an extracellular matrix protein and has been shown to promote adherence of whole bone marrow to extracellular matrix proteins in mice^62^. As this protein may have a role in the bone marrow microenvironment, it is possible that dysregulation of this protein could contribute to the MM pathology. The involvement of DPT in MM pathology needs to be further characterised, such as through mouse models of MM and by exploring whether DPT is dysregulated in the bone marrow in patients with MM. In the current study, MR evidence suggested that higher levels of KDR (VEGFR2) increased risk of MM, and there was no evidence for an effect in the reverse direction (MM risk on levels of VEGFR2). VEGFR2 is involved in endothelial migration and proliferation and is implicated in liver, renal and thyroid cancers, where it is now exploited as a drug target^63^. The role of VEGFR2 in the progression from healthy, through the precursors of MM (MGUS and smouldering myeloma), and to MM, should be further characterised, for instance, by generating proteomic data on patient samples. MR evidence suggested that higher levels of GCLM may result in a decrease in MM risk. GCLM is a subunit of an enzyme involved in the cellular glutathione (GSH) biosynthetic pathway, which is critical to cell survival. Treating MM cells with a proteasome inhibitor, bortezomib (an approved MM treatment), has been shown to lead to higher levels of GCLM. This is directionally consistent with the MR results, where higher levels of GCLM had a lowering effect on MM risk^64^. In addition, there was one protein with evidence of genetic colocalization: uncharacterized family 31 glucosidase KIAA1161, it is unclear how this protein might be involved in MM risk.

Our results point towards putative causal relationships between circulating proteins and MM risk. However, there are limitations to these analyses that need to be considered and results should be interpreted with caution. Firstly, we did not adjust for multiple testing in each individual MR analyses. As we attempted to perform a discovery and replication approach, we believe that adjusting for multiple testing would be too conservative, especially given that the MM GWAS used are not highly powered. Suitable genetic instruments were also not available for all proteins, therefore some potentially important protein-MM or MM-protein effects will inevitably be missed. We used a single *cis*-SNP to instrument circulating protein levels, which limited our ability to perform sensitivity analyses. The assumption of a single causal variant may be overly simplistic, however, approaches using multiple *cis*-SNPs would likely yield similar estimates given that the single *cis*-SNPs used were strongly associated with protein levels (median F statistic of 742 for UKB Olink proteins and 321 for deCODE SomaLogic proteins). Additionally, perturbations in one protein do not occur in isolation. Effects of a single protein may be because of its role in one or more pathways, and therefore it is likely that there is a much more complex interaction between circulating proteins and risk of MM, rather than one or a few proteins being solely responsible for the change in risk. Currently, exploring the contribution of proteins together (as opposed to performing univariable analyses) to MM risk remains a challenge. These analyses were performed in participants only of European ancestry living in the UK, Iceland and Finland, therefore findings may not be generalisable to participants of other ancestries or in other contexts. More highly powered GWAS are required in non-European ancestries in order to evaluate the role of circulating proteins in MM risk more broadly. Another possible limitation is that there may be some misclassification, where participants who were deemed as controls could include those with undetected MGUS or smouldering myeloma, and this may lead to estimates being biased (towards or away from the null).

We identified seven proteins which have consistent MR evidence across two proteomic datasets for a role in MM risk. Some of these proteins have previously been implicated in the other cancer types (VEGFR2 and GCLM), however relatively little is known about these seven proteins in relation to MM risk. Triangulation of evidence across study-designs is crucial to strengthening evidence of association; generating proteomic data from patients with MM (or its precursor conditions) will help to further understand the results observed here.

## Supplementary Information

Supplementary Information.

## Data Availability

All scripts are archived on Zenodo^65^ and available on GitHub^66^. Meta-analysis was performed following the METAL online documentation^44^. All analyses were performed using R version 4.1.2. MR analyses were performed using TwoSampleMR (version 0.4.22)^30^. Colocalisation was performed using coloc (version 5.2.0)^31^. Weighted prevalence was calculated using the *metaprop*() function from the meta package^55^ (version 6.5-0). For the purpose of open access, the author(s) has applied a Creative Commons Attribution (CC BY) licence to any Author Accepted Manuscript version arising from this submission.

## Abbreviations

CRYBB1: Beta-crystallin 1
CRYGD: Gamma-crystallin D
CSF2: Granulocyte-macrophage colony stimulating factor 2
DPT: Dermatopontin
FSTL1: Follistatin-related protein 1
GCLM: Glutamate-cysteine ligase regulatory subunit
GWAS: Genome-wide association study
IL18BP: Interleukin 18 binding protein
IVW-MRE: Inverse variance weighted multiplicative random effects
Kb: Kilobase
KDR: Vascular endothelial growth factor 2
LD: Linkage disequilibrium
Mb: Megabase
MGUS: Monoclonal gammopathy of unknown significance
MM: Multiple myeloma
MMP-9: Matrix metalloproteinase-9
MR: Mendelian randomization
NPX: Normalised protein expression
OBP2b: Odorant-binding protein 2b
PheWAS: Phenome-wide association study
RFU: Relative fluorescence unit
EDTA: Ethylenediaminetetraacetic acid
RSPO3: R-spondin-3
SNP: Single nucleotide polymorphism
TNFSF10: Tumour necrosis factor ligand superfamily member 10
UKB: UK Biobank
